# Professional perspectives on recurrent characteristics of dogs with separation-related problems: a qualitative study in three nordic countries

**DOI:** 10.1038/s41598-026-36791-w

**Published:** 2026-01-21

**Authors:** Emma Almquist, Iben Meyer, Peter Sandøe, Karoline Måseide Thomassen, Ruth C. Newberry, Therese Rehn

**Affiliations:** 1 Department of Applied Animal Science and Welfare, Faculty of Veterinary Medicine and Animal Science, Swedish University of Agricultural Sciences, Uppsala, Sweden; 2 Department of Veterinary and Animal Sciences, University of Copenhagen, Copenhagen, Denmark; 3 Department of Food and Resource Economics, University of Copenhagen, Copenhagen, Denmark; 4 Department of Animal and Aquacultural Sciences, Faculty of Biosciences, Norwegian University of Life Sciences, Ås, Norway

**Keywords:** Separation anxiety, Risk factors, Canine welfare, Behavioural comorbidity, Qualitative research, Thematic analysis, Psychology, Psychology, Zoology

## Abstract

Separation related problems (SRPs) are common in companion dogs and give rise to serious problems for both dog welfare and owner quality of life. However, they are not well understood, making them difficult to prevent and treat. A so far underutilised source of knowledge is experience from professional behaviour practice. In this qualitative study, we explored professionally situated knowledge to identify factors that may contribute to the development of SRPs in dogs. The study is based on 15 semi-structured interviews with a diverse group of dog professionals (veterinarians, animal behaviourists, dog trainers) involved in the treatment of SRPs in Denmark, Sweden, and Norway. The transcripts were analysed using reflexive thematic analysis. Six central themes emerged from this analysis, which were labelled “co-occurring behavioural difficulties”, “a body in flux”, “dog training gone wrong”, “breed type”, “changes to routine and environment”, and “psychological interplay between dogs and their owners”. These themes reflect professional observations of recurring contexts in SRP cases, rather than empirically established risk factors. While some align with existing scientific findings (e.g. comorbidity with noise sensitivity), others highlight underexplored areas, such as effects of adolescence and owner stress. The findings serve to underpin a broad, context-aware view of SRPs.

**Supplementary Information:**

The online version contains supplementary material available at 10.1038/s41598-026-36791-w.

## Introduction

Domestic dogs (*Canis lupus familiaris*) are social animals often kept by humans for companionship^1^. Dogs show a strong attunement to human behaviour and emotion^2,3^ and often use humans to support them in difficult or stressful situations^4,5^. Whereas historically, dogs mainly lived in environments where they had their social needs fulfilled by other dogs or humans (e.g., when living as free ranging village or farm dogs), it has become common to keep companion dogs confined, often with long hours left alone at home^6^. Such management changes have led to an increased scientific interest of separation related problems (SRPs) in dogs^7^, spurring investigations into their manifestations and underlying triggers^8,9^. Still, there remain significant gaps in our understanding of SRPs, including limited use of scientific observations gleaned directly from those who work daily with dogs affected by SRPs^10^.

While the term ‘separation anxiety’ has been the dominant term used to describe SRPs, terms such as ‘separation disorder’, ‘separation syndrome’, ‘separation related behaviours’ and ‘separation related problems’ reflect the multifaceted nature of behavioural responses to intermittent separation^9^. SRPs in dogs is argued to represent a syndrome rather than a clear diagnosis^11^. Hence, we refer to SRPs to denote all facets of a dog’s behaviour that are reported as problematic by the owner in the context of repeatedly being left alone or separated from one or more specific people with whom the dog has a close relationship. Fundamentally, SRPs are characterised by various behaviours (destructive behaviour, vocalisations, inappropriate elimination etc) that manifest in the actual, perceived, or imminent absence of the owner or other significant people^10^. Different dogs may display different combinations of these behaviours, and they can recur with varying frequency, from occasionally to every instance the dog is left alone^11^. Some symptomatic behaviours (e.g., passivity) may be overlooked by owners^11^, making SRPs difficult to identify in dogs and compare across studies.

SRPs can present a significant threat to the welfare of dogs and the individuals who care for them. Dogs with SRPs not only face considerable emotional distress but also have an increased likelihood of being relinquished or euthanised^12^. Moreover, treating SRPs can be challenging, a sentiment expressed by 31% of animal behaviour counsellors participating in a Swedish survey^13^. Challenges sometimes stem from the complexities of recommended interventions. For instance, when dog owners attempt behavioural interventions, they might not adhere to the recommended procedures due to the emotional strain of having seen their pets in distress during separation, or difficulty in complying with the demands of the techniques themselves^14^. The treatment of SRPs can be further complicated by the presence of other behavioural concerns such as generalised fear and aversion to loud noises^8,15^. Furthermore, dogs with SRPs tend to exhibit a more “pessimistic” cognitive bias compared to dogs without such problems^16,17^, suggesting a lower quality of life.

Investigations into the prevalence of SRPs in dogs based on convenience samples have yielded varied estimates (Finland: 5–18%^8,18,19^; Norway: 20%^20^; UK: 34%^21^). A Danish survey of citizens, using methods aimed at acquiring a representative sample of dog owners, reported a prevalence of only 4% in Denmark^22^. Owner-reported prevalence based on convenience samples often creates selection bias towards owners most concerned about their dogs’ behaviour. Yet even representative surveys are constrained by owners’ varying ability to recognise symptomatic behaviours. Regardless of the frequency of SRPs in the dog population, there is no denying the significance of SRPs, which have been reported as the second most common reason (after aggression) for dog owners seeking assistance from a behaviour specialist^23,24^. The reported figures emphasise the importance of recognising, understanding and addressing these problems for the well-being of both dogs and their owners.

Although SRPs are widely recognised, their underlying mechanisms remain poorly understood, and no definitive diagnostic test currently exists^11^. This lack of clarity hampers both prevention and treatment efforts. A valuable yet underutilised source of insight lies in professional behavioural practice. Addressing this gap, the present qualitative study engages directly with professionals involved with dogs and their owners to integrate practice-informed perspectives^7,11^ and build on previous research on behavioural correlations and predispositions, including associations between SRPs and specific phobias^15,25^. Specifically, it aims to (1) identify dog characteristics and backgrounds commonly observed by professionals in SRP cases and (2) assess how these experiences align with established risk factors in the literature.

## Results and discussion

**Fig. 1:**
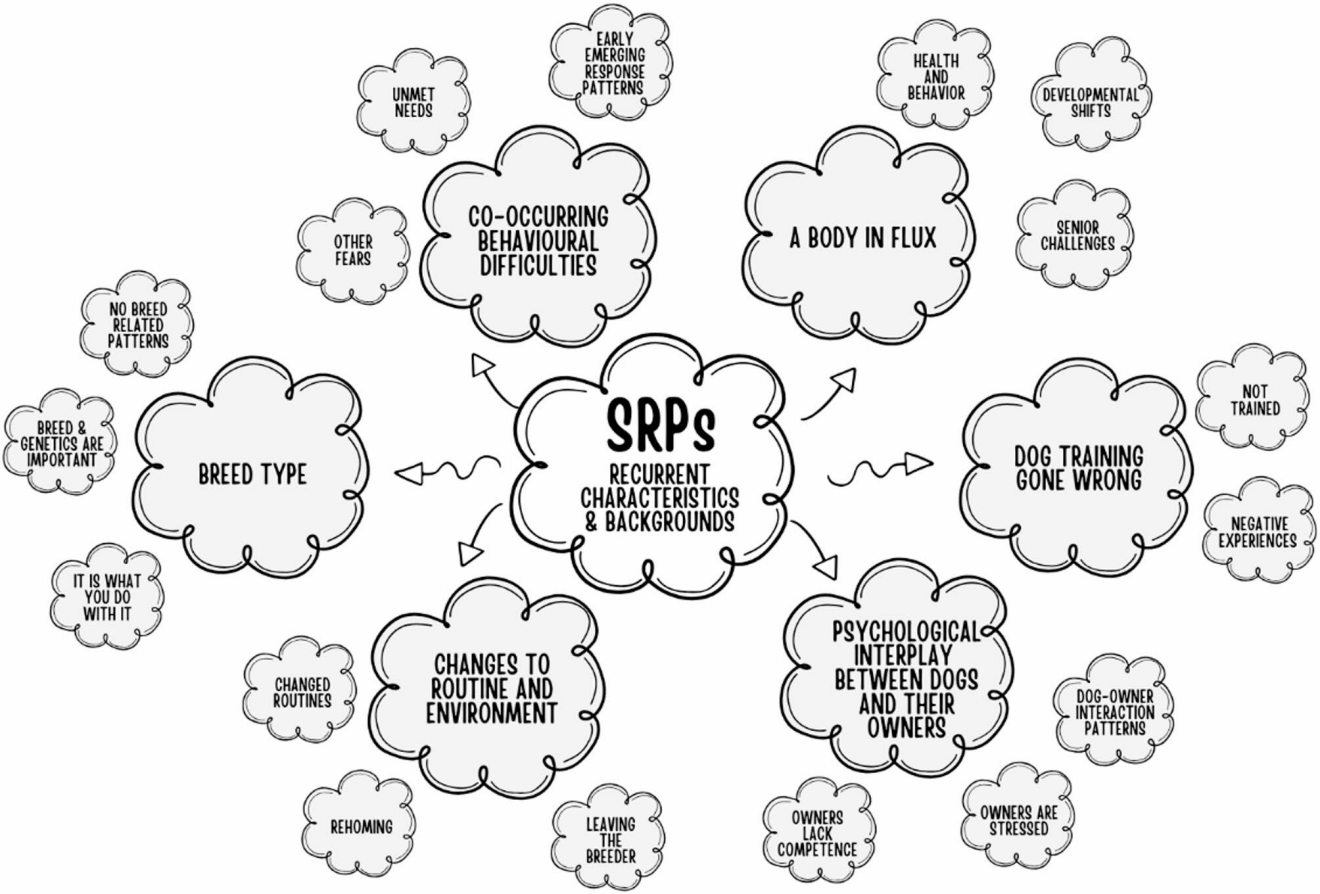
An overview of the six central themes and their sub-themes, derived from the reflexive thematic analysis. Some names are shortened in this figure.

This section presents an integrated analysis of the themes identified through interviews with dog professionals across Sweden (*n* = 5), Denmark (*n* = 5), and Norway (*n* = 5). Drawing from reflexive thematic analysis^26^, we highlight six central themes, each with several sub-themes, capturing frequently reported backgrounds and characteristics of dogs with SRPs (Fig. 1; see Supplementary information 1 for quotes). Each theme is accompanied by interpretive insights grounded in current literature, situating professional observations within a broader scientific context and highlighting their potential implications for canine welfare, training, and preventive interventions.

### Theme 1: co-occurring behavioural difficulties

Statements about “co-occurring behavioural difficulties” reflect how behaviour problems in dogs with SRPs are often interconnected. Dog professionals commonly reported that dogs with SRPs also showed sound sensitivities, specific fears, stress-related behaviours, resource guarding, and reactivity on walks.

#### Other fears

This sub-theme reflects the reported fear and anxiety expressed by dogs outside of a separation context that might affect a dog’s stress levels and behaviour when left alone.

“But if a dog has issues in several areas and that’s not unusual really, I would say, then that affects the separation training too, and it has to do with all the impressions that need to be processed [by the dog] and then suddenly [the dog] has to be alone with all - everything it has experienced, in a way.” ID4NO.

The most commonly noted comorbidity in this current investigation and in previous studies was between noise sensitivity and SRPs^20,27^. Noise sensitivity is one of the most common canine anxieties^19,22^, but it is unclear if it is causally related to SRPs.

#### Early emerging response patterns

This sub-theme represents respondents’ reflections on whether innate or early differences in behavioural responses, such as heightened anxiety and/or low stress resilience observable from early puppyhood, may influence SRPs.

“There are some that have had it [an SRP] from the time they were puppies, who have never been able to be alone. Never have been able to be alone at home, where owners say that from Day 1 they have never been able to leave [the dog], even just to go to the toilet, and that they have never been able to leave, go out the door for even 10 seconds, so they have never felt that they could train anything at all related to [the dog] being alone at home.” ID2DK.

Dogs displaying SRPs have been found to be more impulsive, inattentive, excitable, fearful, anxious, neurotic, or to exhibit a negative cognitive bias^17,19,28,29^. This might be because such dogs are more prone to stress and negative emotions^29^ and/or because living with an SRP induces negative affective states^30^.

#### Unmet needs

Frustration and boredom, arising from unmet physical or mental stimulation needs, were identified as factors that can exacerbate or mimic SRP symptoms.

“And then I have some [dogs] … where it’s not really separation anxiety, but where they are under-stimulated and so when they are alone, they find a lot of things to do because they have a hard time relaxing in that situation.” ID4SWE.

In a Finnish study, daily exercise was identified as the strongest environmental factor differentiating dogs without and with SRPs^18^, suggesting that insufficient physical activity may contribute to the problem, perhaps due in part to boredom. Boredom may, in turn, be associated with frustration, with some symptoms of SRPs such as excessive barking being expressions of this state^31^. In their review, Meneses et al.^30^ proposed that if frustration is a causal mechanism for SRPs, dogs should exhibit similar frustration-related behaviours also in the presence of their owners. However, as behavioural topographies often vary depending on context, this assumption warrants further studies.

### Theme 2: a body in flux

In this theme, dog professionals highlighted how physiological changes and health issues were considered to influence SRPs in dogs. The respondents often noted that dogs with SRPs were undergoing developmental transitions (puppyhood, sexual/social maturity, aging), or living with health conditions, and emphasised the need to integrate physical health with behavioural observations.

#### Early developmental shifts

This sub-theme highlights adolescence as a vulnerable period, when physiological and psychological changes may trigger or worsen SRPs.

“But I see a connection between [SRP s] and, somehow, developmental periods. Often when the puppies - the young dogs - are in an easily spooked period at 6–8 months, it can happen that suddenly they can’t be left alone (even the young dogs). It’s when they are 18 months - there somewhere - [that] they usually develop problems.” ID2SWE.

In a survey of 13,715 Finnish dog owners, Salonen et al.^19^ found no consistent linear trend in SRP prevalences across six dog age groups (10 weeks-2 years, 2–4 years, 4–6 years, 6–8 years, 8–10 years, 10–18 years). However, they did not investigate the timing of emergence of SRPs in the youngest age period. The dog professionals reported an impression that the risk of developing an SRP is elevated during adolescence, and suggested that sexual and social maturation may trigger SRPs even in previously calm dogs, pointing to a need to study hormonal influences. It has been proposed that dogs, like humans, may experience a “teenage phase” when going through puberty, characterised by increased attachment-related anxiety^32^. Lord et al.^33^ reported SRPs in 5–8% of dogs at 6–9 months, at the lower end of the reported prevalences among the general dog population of 4–34%^8,18–22^. Notably, the dog professionals in this study referred to dogs aged approximately 6–18 months, a broader window than Lord et al.’s 6–9 months^33^. Moreover, they^33^ also reported lower rates of all problem behaviours than reported in other studies, likely because many issues at that age are not yet severe or established enough to be recognised by owners. Thus, while owners may not label an SRP during early adolescence, professionals later consulted can often trace onset to that period. It could also be that there is a heterogeneity of aetiologies lumped under the SRP banner^11^, with each functional condition having a different prevalence and development timescale.

#### Senior challenges

This sub-theme denotes that senior dogs are prone to developing SRPs, even if they have shown no signs earlier in life.

“[…] and also in older dogs. Then, of course, since we know that it can be linked to cognitive dysfunction or dementia and similar conditions.” ID5SWE.

The respondents reflected on how declining cognition may affect SRPs, although Salonen et al.’s survey indicated no rise in prevalence in their oldest age group as a whole^19^. Reports on aging and cognitive decline in dogs have mentioned general fear and SRPs as common symptoms^34,35^ and Mongillo et al.^36^ speculated that this may be due to a decrease in the ability to cope with social isolation.

#### Health and behaviour

This sub-theme exposes differing views on health and SRPs, whereby some respondents saw bodily discomfort as a key factor, while others did not observe a clear link.

“If something is pain-related or, as I mentioned earlier, something like itching and allergies or something going on with the intestines or stomach, clearly, it affects the dog’s response very much.” ID4NO.

“At least I haven’t found anything where they have looked into whether dogs with separation related problems often are in pain. […]. So, the answer to that is, well, it’s not the first thing I think about.” ID2SWE.

Physical illness can trigger SRP relapses or worsen symptoms in affected dogs, while chronic SRPs may contribute to illness^37^. Hence, when an SRP appears without a clear trigger, medical causes like gastrointestinal issues or cognitive dysfunction should be considered^37^. Hormonal changes were also mentioned by professionals as potential bodily contributors to SRPs. Research on neutering and SRPs is inconclusive, as timing is rarely accounted for^30^. One study found lower SRP rates if dogs were neutered before the age of 5.5 months^38^, but neutering may also increase noise fears linked to SRPs^38–40^, pointing to the need for further investigations^41^.

### Theme 3: dog training gone wrong

“Dog training gone wrong” concerns how early training, especially around being alone, impacts the development of SRPs. Professionals consistently identified poor or absent early training as a common background in dogs with SRPs, particularly among dogs used to constant companionship, such as “Covid dogs” (e.g. dogs acquired during the Covid-19 pandemic and lock downs).

Respondents underlined that SRPs are often preventable through informed training. Notably, no scientific studies were found on protocols for teaching dogs to be alone, regardless of whether or not dogs already show SRPs, which underscores the need for future research in this area.

#### Not trained

This sub-theme focuses on respondents’ views on how lack of early training can lead to or worsen SRPs, often due to owner inexperience, misconceptions or lifestyle factors such as those related to the Covid-19 pandemic.

“A common factor, or a quite common factor, is that they haven’t even trained [the dog] to be alone when they seek help but have just realised [the need]. And especially during the pandemic, it was very common that they never needed to leave the dog and then suddenly the dog is 2 years old, and they realise that they might need to start training [the dog] to be alone and then they haven’t laid any groundwork for it.” ID4SWE.

Well-socialised and ‘gradually trained’ puppies have been found to handle separation better than untrained puppies^42^. A study found that 10% of the previously unaffected dogs in their study developed an SRP after the Covid-19 pandemic^43^, likely due to not having been left alone to the same extent during the pandemic and therefore lacking a gradual increment in experience of being alone.

#### Negative experiences during training

Respondents warned that rushed or forceful training can worsen SRPs. Letting dogs “cry it out” may reduce symptoms without diminishing stress. They recommended what they described as “gradual” and “welfare-based” methods instead.

“I would also say that one characteristic is extremely unrealistic [owner] perceptions, and that they get them from Dr. Google - Many get [ideas] like this: leave the room when the dog sleeps. Can you imagine anything more cruel than you go to sleep, and then everyone’s gone when you wake up?” ID5NO.

Such statements illustrate professional frustration with misinformation but also highlight how professional interpretations may be shaped by expectations regarding appropriate owner behaviour and knowledge. In this context, it is notable that SRP-specific studies comparing the efficacy of different reinforcement and punishment-based learning contingencies are lacking. However, aversive training methods have been linked to increased fear, stress, and behavioural problems^33,44,45^ which may in turn be linked to SRPs (see Theme 1). It has been suggested that common advice on separation training may miss key stress-reducing elements, like predictability and frustration tolerance^46^. At present, best practice protocols for gradual, welfare-friendly training have not yet been established in scientific literature.

### Theme 4: breed type

Almost all participants mentioned breed type in relation to SRPs, though opinions varied on its importance. This theme underscores that while breed is frequently considered by professionals, the link between breed and SRPs is complex, shaped by genetics and environmental factors such as owner behaviour. Respondents emphasised that upbringing and training play a major role and that breed traits alone do not determine whether a dog will develop an SRP.

#### Breed and genetics are important

This sub-theme acknowledges the role that genetics and breed-specific predispositions have in influencing susceptibility to SRPs.

“There is presumably some genetic factor also, that matters here in terms of anxiety at least” ID1DK.

“Companion dogs are bred to be together. They take it [separation] much harder. Interestingly, we have some breeds that aren’t necessarily bred to be companion dogs but still have it [an SRP]. For example, we know the Dachshund can be very prone to SRPs. So yes, breed definitely plays a role.” ID2NO.

Even though there has been some controversy relating to this issue, there is good evidence showing links between breed and dog behaviour and personality^47,48^. There may also be differences in behavioural dispositions within breed sub-groups, for example between show and utility lines as well as within sub-group individual differences. Research supports genetic links to fearfulness and SRPs^49–51^. Some breeds, like Cocker Spaniels, Schnauzers, and Dachshunds, have been overrepresented in SRP cases^52^. Moreover, cooperative working dog breeds have been reported to show more arousal-related SRP behaviours, such as barking and restlessness when alone^53^.

#### There are no breed related patterns

This sub-theme reflected that some professionals did not consistently observe clear links between specific breeds and SRPs.

“What I can see, is that I can’t see that there’s any breed-specificity - I can’t see that I have specific breeds.” ID5DK.

Previous studies have found that mixed breeds have a higher percentage of SRP-affected dogs than dogs of any single breed^19,49,54,55^. However, Meneses et al.^30^ reasoned that since shelter dogs are often overrepresented in studies on SRPs, the high percentage of mixed breed dogs in shelters might overshadow patterns across pure bred dogs. If different forms of SRPs arise from different mechanisms, it will be necessary to tease them apart with precisely defined diagnostic criteria to better delineate their respective genetic underpinnings.

#### It’s what you do with the breed that matters

This sub-theme highlights how the environment, including training and owner behaviour, can strengthen or reduce breed-related tendencies.

“I think genetics absolutely play a role, but I think there are very many environmental type things that are at least as important, if not more.” ID3NO.

Both respondents and scientific reports emphasise that SRPs result from a mix of heredity and environment. Heritability of behavioural traits is often relatively low, though robust among-breed heritabilities between 0.4 and 0.5 have been reported for SRPs^56^. Nevertheless, environmental factors strongly influence adult dog behaviour^57^. Small dog size has been linked to behaviour issues like SRPs^58^, but Tiira & Lohi^18^ found this only after excluding daily exercise and suggested that routine, not size, may be a key factor.

### Theme 5: changes to routine and environment

The theme “changes to routine and environment” describes how major disruptions, such as rehoming, changes in daily life, or leaving the breeder, can trigger SRPs by undermining a dog’s sense of safety and stability. Respondents emphasised that dogs rely heavily on routine to feel secure when alone, and that changes in social or environmental context often lead to stress, anxiety, and SRPs.

#### Changed routines

This sub-theme reveals how changes like moving, divorces, or new family members can unsettle dogs, with respondents noting that disruptions in routine and changes in the social environment may trigger or worsen SRPs.

“Yes, I think it’s that suddenly things have changed, like routines. That they [the dogs] have moved, even if it’s with the same family that they have moved. That they [the owners] have divorced so that the dog just lives in one place with half the family, so to speak. And changed work hours […] can affect it [the SRP]. A change in the family if they have had children – such things can also affect the dog.” ID2SWE.

Changes in routines have been proposed as risk factors for SRPs in other studies^28,59^. Divorce, in particular, seems to increase attention seeking behaviour in dogs, which may in turn be linked to the development of an SRP^60^.

#### Rehoming

This sub-theme highlights a possible vulnerability of rehomed dogs to develop SRPs, as respondents reported that rehomed dogs are common within the population of dogs they see with SRPs.

“I see connections with these rescue dogs or rehomed dogs, as I mentioned, that bond very closely with their owners and find it difficult to be away from them.” ID1SWE.

While some studies have found no difference in SRP prevalence^49,61^, others have reported high SRP rates among adopted shelter dogs (79–88% of adopted dogs^62,63^). No clear cause has been established, but contributing factors may include shelter-related stress^30^, severance of contact with the previous owner, a preexisting SRP or other behaviour problem(s), and insufficient socialisation and training.

#### Leaving the breeder

This sub-theme encapsulates discussions about the significant impact of the transition from the breeder to a new home on a dog’s ability to cope with separation in the future.

“Early separation from the mother dog, or if a puppy became sick and needed to be removed or the mother dog got sick and was removed. Or the last puppy left in the litter who has been with the mother dog a lot, there I can see a certain tendency.” ID1SWE.

Results from several studies^18,64,65^ suggest that early experiences in the puppy den and the transition to a new owner impact a dog’s ability to handle stress. For example, it has been found that dogs with multiple anxieties, including SRPs, often were reported by owners to have received lower maternal care^18^. In Denmark, Sweden and Norway, legislation prohibits breeders from selling puppies before they reach 8 weeks of age. Weaning earlier than this has been associated with a raft of problematic behaviours including symptoms of SRPs^64^.

### Theme 6: psychological interplay between dogs and their owners

Within this theme, we describe respondent views regarding how the owner’s mental state and the quality of the dog-owner relationship and interaction patterns can influence the development of SRPs. Dog professionals frequently identified owner characteristics as a common background factor in dogs with SRPs, suggesting that emotional interdependence between dogs and owners plays a key role.

#### Owners are stressed

This sub-theme relates to how dogs may mirror their owners’ anxiety, suggesting that treating SRPs could also require supporting the owner’s mental health.

“I also experience … when I start talking about stress in the dog, then they say ‘Oh no, I know what you’re talking about because I’ve been off work sick with stress.’ So, I often experience that when we start to get into it [the dog’s stress], then the owner has also often been … exposed to stress, and has maybe had a breakdown due to stress” ID3DK.

Respondents observed that dogs with SRPs often have anxious owners, a link partially supported by previous findings. Dogs may reflect their owners’ acute stress^66^, and studies using hair cortisol suggest long-term stress synchronisation, likely due to dogs mirroring owners’ stress levels^67^. While one study connected owner neuroticism to similar traits in their dogs such as being easily upset^68^, another found no link between owner neuroticism and SRPs^29^.

#### Owners lack competence

This sub-theme stresses that lack of knowledge and unrealistic expectations about dog care, especially separation training, can contribute to SRPs. Educating owners was highlighted as important for improving welfare for both dogs and humans.

“But that they don’t expect to have to spend a lot of time on them [dogs], I think that’s a recurring theme. That people had a bit of a wrong understanding of what they were getting into when they got a dog.” ID3NO.

Several studies have reported that attending dog classes was linked to reduced SRP risk^33,54,59,69^, though it is unclear if this was due to improved socialisation, improved owner skills or both^30^. While prior dog experience may help set realistic expectations, it does not necessarily prevent behaviour issues^33^.

#### Dog-owner interaction patterns

This sub-theme covers how specific qualities in the dog-owner bond, common in SRP cases, may influence the dog’s behaviour.

“[…] there are some [cases] where […] the dog is obviously very attached to one owner, where there’s a big difference depending on whether it’s one owner leaving […] or the other owner, and if one owner disappears from the home, then the dog also howls.” ID2DK.

The respondents noted that close bonds, sometimes referred to as “hyper attachment”, may increase SRP risk. While commonly mentioned in behaviour texts, “hyper attachment” lacks a scientific definition and is not formally recognised in attachment theory^70,71^. Most studies do not find a strong link between SRPs and the quality of the dog’s attachment to the owner^59,70,72^. However, some evidence suggests that dogs with more owner interaction, such as play, show fewer SRP symptoms^28,49^.

### Additional note

One informant (ID1NO) answered that they did not see any reoccurring characteristics or background characteristics associated with SRPs within the population of dogs they met with SRPs.

### Strengths and limitations

This study applied reflexive thematic analysis to explore dog professionals’ perspectives on SRPs. A key strength is that it brings to the fore practice-based insights that are seldom captured in quantitative studies, thereby contributing a dimension based on professionally situated knowledge to the evidence base. The inclusion of respondents from three Nordic countries, as well as veterinarians, trainers, and behaviourists, enhanced the diversity of views represented. Reflexive thematic analysis also facilitated a transparent and iterative analytic process, supported by researcher reflexivity.

Several limitations must be noted. First, the sampled respondents may not reflect the broader population of practitioners: respondents were recruited through professional networks, and unevenly distributed across professional roles, with most respondents identifying as behaviourists (*n* = 11) and/or trainers (*n* = 9), and fewer as veterinarians (*n* = 4; two of whom also identified as behaviourists). The number of participants was influenced by practical time constraints, as conducting and analysing qualitative interviews is time intensive. However, the number of participants is consistent with common practice in qualitative research using semi-structured interviews, where sample sizes typically range from 10 to 30^73,74^. Moreover, prior research indicates that thematic saturation can often be reached within 12–15 interviews^75^. While saturation was not used as an analytic stopping criterion in this reflexive thematic analysis, the present sample was considered sufficient to capture the key themes relevant to the research question. Second, interviews were conducted in three languages and later translated for analysis, which may have altered nuance, despite careful editing. Third, the interview guide included examples of possible backgrounds and characteristics, which could have shaped responses toward pre-identified topics. Fourth, themes were primarily coded by one researcher, and although reflexivity was emphasised, inter-coder validation was not undertaken. Finally, the study reflects qualitative professional perceptions and should, thus, be regarded as exploratory and hypothesis-generating rather than confirmatory.

### Implications and future research

The findings in this study emphasise the value of practitioner insight when it comes to understanding and preventing SRPs. We identify several dog experiential backgrounds and characteristics that could be common for dogs with SRPs, pointing to possible risk factors, such as adolescence, hormonal changes, and lack of early training, that were mentioned by the respondents and deserve more research.

In addition to highlighting areas where practitioners foreground factors that remain underexplored in the literature, the present findings also point to the converse pattern: several scientifically discussed SRP perspectives and risk factors were only rarely articulated by respondents. Respondents seldom noted that associations may be bidirectional, such as behavioural characteristics being either predispositions or consequences of living with SRPs^29,30^, and SRPs both exacerbating illness and being exacerbated by illness^37^. Maternal care quality, previously linked to multiple anxieties including SRPs^18,64,65^, was also not explicitly identified within the context of early-life factors. Finally, while respondents described strong owner-directed bonds using terms such as “hyper attachment”, their accounts did not reflect that “hyper attachment” lacks a scientific definition and is not formally recognised in attachment theory^70,71^. Together, these patterns may suggest gaps in knowledge translation between research and practice, variability in how routinely practitioners engage with the research literature, or a primary focus on factors that can be addressed in adult dogs presenting with SRPs.

We also note that inconsistent definitions and measures of SRPs make comparisons difficult, emphasising the need for interdisciplinary collaboration to standardise terms and tools. Future research should aim to examine causal links involving health, attachment styles, and early experiences, and develop evidence-based prevention protocols. Overall, our findings call for a broad, science-informed approach that connects research with real-world practice.

Participant gender was not included in the analytic framework to preserve anonymity and because gender was not a primary focus of the study. However, we acknowledge that professional perspectives on training practices, dog-owner relationships, and interpretations of stress may be shaped by gendered norms and roles within the field. Future qualitative work could explicitly explore how gendered subjectivities and power relations influence professional discourse around SRPs.

## Conclusions

This study examined common backgrounds and characteristics of dogs with SRPs, based on insights from dog professionals in Sweden, Denmark, and Norway. Using reflexive thematic analysis, six themes were identified, highlighting behavioural, physiological, environmental, and relational risk factors. Findings show that SRPs were frequently reported to co-occur with behavioural difficulties and to be influenced by factors such as the dog’s physical condition, training practices, changes to routine and environment, as well as the emotional dynamics between the dog and its owner. The role of breed in relation to SRP was described as both relevant and irrelevant, reflecting divergent perspectives among respondents.

## Methods

We conducted semi-structured interviews with dog professionals in Denmark, Sweden, and Norway, targeting a diverse group of professionals within the behavioural field: veterinarians, behaviourists specialised in handling challenging dog behaviours and dog trainers responsible for general training programs^76^. Eligible participants were those actively engaged in dog behaviour management and specifically advising on SRPs in dogs on a regular basis (i.e., having at least one year of regular professional experience in advising and assisting owners of dogs with SRPs, including the current year).

A list of potential interviewees was compiled through professional networks and online research. When contact was initiated, potential participants were informed about the purpose and format of the study and asked about their professional background and current work to determine if they were eligible to participate in the study. In Sweden, all five dog professionals who were contacted agreed to participate. In Denmark, seven dog professionals were invited but one declined, and another did not respond. In Norway, 11 dog professionals were contacted of whom five met inclusion criteria and agreed to participate.

Prior to interviews, participants received detailed information sheets via email, outlining interview themes, data handling, and anonymity assurances. The informants were encouraged to ask questions before the interview and gave their informed consent verbally at the beginning of the recorded interviews. Communication was conducted in each participant’s native language to enhance understanding and ensure nuanced information.

In total, we recruited five interviewees from each country (15 in total, see Supplementary information, Table S1). The respondents were categorised based on definitions from Daniels et al.^76^, who state that dog trainers provide general training while behaviourists are trained to deal with problematic behaviour. Most respondents represented more than one professional category; four participants were veterinarians, 11 were behaviourists, and nine were dog trainers.

### Ethical considerations

This study was a part of a larger research project entitled “Treatment of separation related problems in dogs: what works and what does not”. This larger project received ethical evaluation regarding the researchers involved, research protocols, data processing agreement, interview guide, informed consent and general information provided to the participants, and was approved by The University of Copenhagen Research Ethics Committee for Science and Health (Reference number: 504 − 0431/23–5000). In addition, approvals were obtained for participants in Sweden (Swedish University of Agricultural Sciences, Reference number: SLU.thv.2025.2.2–336) and Norway (Norwegian Agency for Shared Services in Education and Research; Reference number: 577407). The study was conducted in full compliance with the relevant guidelines and regulations, as specified in the approved ethical permits.

### Interviews

The interview guide encompassed broad questions designed to evoke reflection by participants regarding commonly observed contexts in which separation-related behaviours occur (see Supplementary information). The purpose for asking each question was specified. Interviews with participants were conducted via Zoom videoconferencing (Version 5.16, Zoom Communications, Inc., San Jose, CA, USA) by one interviewer per country and language. Prior to interviews, the three interviewers met online and carefully reviewed the guide to ensure similar implementation of the interviews. Thereafter, pilot interviews were conducted and necessary edits were made to the interview guide. Each interview took about 45 min to one hour. Following the interviews, audio files were extracted, and video files were deleted.

Auto-transcription of the audio files was performed in NVivo 12 (Version 14.23.0; developed by QSR International, now part of Lumivero, Burlington, MA, USA; lumivero.com/products/nvivo), with final editing by the respective country interviewer. The first author translated the transcripts to English. The audio files were stored securely and coded for privacy, and the transcripts were edited to make each respondent anonymous before sharing them with other project participants. Analysis commenced once all transcripts were anonymised and translated.

### Thematic analysis

We adopted a reflexive thematic analysis approach, adhering to the six-stage framework^26^. Behaviour is inherently complex, and reflexive thematic analysis offers the analytical depth necessary to grasp nuances, complexities, and contradictions within the data. This study specifically employed an inductive variant of reflexive thematic analysis, prioritising data-driven analysis and letting the transcript content lead the analysis. This approach allowed us to develop themes organically from the data, ensuring that any analysis remained grounded in the actual content of the data rather than preconceived notions or existing literature on the subject.

In the initial stage of analysis (“Familiarising yourself with the dataset”), the first author immersed herself in the data, conducting thorough readings of each transcript to develop an intimate understanding of the content. This involved printing out the transcripts for detailed, handwritten annotations. The second stage (“Coding”) involved generating initial codes. These preliminary codes were recorded both within the margins of the transcripts and on separate sheets to facilitate an overview of emerging patterns. The third through fifth stages (“Generating initial themes”, “Developing and reviewing themes” and “Refining, defining and naming themes”) focused on finding patterns between the initial codes and developing themes. This involved multiple re-readings of the data and an iterative process of sorting, combining, and subdividing codes. Preliminary themes were then formed, carefully labelled to capture their essence and relevance. To ensure robust data support, particular attention was given to the prevalence of specific themes raised independently by different respondents. In the sixth and final stage (“Writing up”), a detailed analytical report was developed, linking the refined themes directly to the research inquiry. The themes were described in a narrative style, with selected quotations serving to illustrate key points and enhance thematic descriptions. The choice of style was made following the recommendation by Braun and Clarke^26^ to ensure an engaging and accurate representation of the theme. Efforts were made to balance the representation of respondents’ views to prevent the overrepresentation of any single viewpoint. For each chosen quote, the associated respondent code was noted. After selecting all quotes, they were reviewed for any overrepresentation of specific respondents. In case of overrepresentation, quotes were revisited to achieve improved balance without compromising the integrity of the theme’s representation.

The methodology during the theme development and presentation of results was experiential, aimed at capturing the informants’ personal perceptions of SRPs. This approach prioritised the authenticity of the informants’ professionally situated knowledge, providing a direct reflection of their views within the thematic analysis.

### Researcher reflexivity statement

Within the recommended practice for reflexive thematic analysis is a written statement on the first author’s (EA) background and possible associated biases^26^. EA’s path to researching canine SRPs is deeply rooted in both professional- and personal experience. With a Bachelor’s degree in biology, a Master’s degree specialising in ethology, and eight years dedicated to educating dog owners about animal behaviour through courses, lectures and private consultations, she has accumulated both academic knowledge and practical experience. EA’s studies and experiences have coloured her understanding of the issue, and she did not enter this project without preconceptions. She did, however, approach the subject with a curiosity fuelled by gaps in scientific knowledge she had noticed during both academic and applied, practical work. Her personal journey with her own dog’s SRP lent her an insider’s perspective on the challenges and strategies involved in managing this condition from the perspective of a dog owner. In addition, EA has worked on the treatment of several SRP cases in her professional role. She views SRPs as a serious welfare risk for both dog and owner. Engaging in qualitative research for this study marks a significant shift from her quantitative background, motivated by a curiosity to explore nuances of dog behaviour as reported by dog professionals.

### Use of LLM

Chat GPT-4 (OpenAI, San Francisco, CA, USA; chat.openai.com/) was used to suggest names for themes that emerged from the analysis and to double check translations. All outputs were reviewed and edited by the authors, who take full responsibility for the final text.

## Supplementary Information

Below is the link to the electronic supplementary material.

Supplementary Material 1

## Data Availability

Anonymised interview transcripts are available to researchers upon reasonable request. To obtain access, please contact Therese.Rehn@slu.se. Access will be granted on the condition that an agreement not to share the data is signed.
