# Supplementary material for: Professional perspectives on recurrent characteristics of dogs with separation-related problems: a qualitative study in three nordic countries
Source: Sci Rep. 2026 Jan 21;16:2627. doi: 10.1038/s41598-026-36791-w (PMC12824252; doi:10.1038/s41598-026-36791-w)
Supplement: Supplementary file 1 — Supplementary Material 1 [file 41598_2026_36791_MOESM1_ESM.pdf]

Supplementary information 1

Content:

- Table S1 (description of professional roles)
- Interview Guide
- Quotes divided by theme

Table S1. *A description of the respondent's professional roles, as defined by Daniels et al. (2023)*

	Veterinarian	Behaviourist	Trainer
**ID1SWE**		X	X
**ID2SWE**		X	X
**ID3SWE**		X	
**ID4SWE**		X	X
**ID5SWE**	X		
**ID1DK**	X	X	
**ID2DK**	X	X	
**ID3DK**		X	
**ID4DK**			X
**ID5DK**		X	X
**ID1NO**		X	X
**ID2NO**		X	X
**ID3NO**	X		
**ID4NO**		X	X
**ID5NO**			X

Interview guide

Qualitative interview overview of population and recruitment

15 interviews carried out in Denmark, Sweden, and Norway, 5 in each country.

This guide was translated into Danish, Swedish and Norwegian and the interviews were conducted in mother tongue.

<u>Inclusion criteria of informant population:</u>

1) Working professionally with giving advice to dog owners about behaviour related problems
2) At least one year of experience with cases of separation related problems

<u>Minimum quota</u>

At least 5 professionals from each of the three countries
At least 1 professional from each country with a veterinary background (vets*)
At least 1 professional from each country who is specifically trained to work with undesirable, problematic and/or dangerous behaviour (behaviourists*)
At least 1 professional from each country who plan and manage the general training of dogs for example in group classes (trainers*).
*Terms and definitions taken from https://doi.org/10.1016/j.jveb.2022.11.011

Objective of this part of the research project: to describe treatment strategies and experiences among animal behaviour professionals treating SRPs

Objective of this interview: to gather information from representatives of different groups of professionals that work with SRP- treatment. The information will form the basis for developing a questionnaire about SRP treatment for a larger population of dog professionals.

According to the project description, the interviews and the following questionnaire "*will collect <u>information about strategies currently being used to treat SRPs</u> and identify <u>patterns of preferred strategies as well as common challenges</u> seen from consultants' point of view."*

Parts in the interview guide (part 7 was the main focus related to the article entitled 'Professional perspectives on recurrent traits in dogs with separation-related problems: a qualitative study in three Nordic countries'):

Part 0: Welcome, information, consent – max 3 minutes

Part 1: Background and current (behaviour) work situation – max 3 minutes

Part 2: Defining SRP cases – max 3 minutes

Part 3: Defining successful and unsuccessful treatments – max 3 minutes

Part 4: Successful SRP treatments (what worked well) – max 8 minutes

Part 5: Unsuccessful SRP treatments (what wasn't working) – max 8 minutes

Part 6a: Current strategy/strategies for SRP treatment - max 10 minutes

Part 6b: Non-training treatments – max 3 minutes

Part 7: Recurring dog characteristics and treatment adaptations – max 5 minutes

Part 8: Owner management, expectations, compliance – max 3 minutes

Part 9: Information acquisition about the treatment of SRP – max 3 minutes

Overall duration: approx. 60 minutes

INTERVIEW GUIDE

Questions in the left-hand column are approximate questions to be used by the interviewer. Text in square brackets and underlined i.e. <u>[xxx]</u> is information to the interviewer (should not be read out to the informant). Text in right-hand column explains the purpose of the part/questions.

**Part 0. Welcome, information, consent**	
Thank you for agreeing to participate in this interview. Before we start, have you read the information we have sent you? Do you have any general questions about the information? I will be recording this conversation and saving the audio file in accordance with the information you've read. Do you have any questions regarding this?<br><br>[if needed, read the information below, which has also been given in the information sheet]	Introduction
The interview is part of a research project about separation related problems in dogs focusing on which treatment strategies seem to work best. The aim of today is for me to gather as much information as possible about your experiences with working with separation related problems in dogs. We would like to learn about both your good experiences as well as any challenges you have experienced in this work.<br><br>*The interview should take no more than one hour. It will be semi-structured, so I have some broad questions that I want to answer, but I will try to reach the answer through an informal conversation where your perspective can help shape the questions. You can always decline to answer any question, and you are very welcome to ask about the reason for a specific question or anything else.*<br><br>*I will be recording our conversation. The audio of the recording will be transferred to the data responsible researcher at the University of Copenhagen and the video files will be deleted. I will make an anonymized written version of the interview, a so-called transcription, which will be used for my master project at Sveriges lantbruksuniversitet as well as for a Norwegian master project at Norges miljø- og biovitenskapelige universitet* <u>*[this sentence, and the next one, are adapted to fit each interviewer].*</u> *These transcriptions of the interview will therefore be shared between me, my supervisor, the Norwegian master student and her supervisor as well as the data responsible researchers at the University of Copenhagen. After the transcription, I will delete the audio files and these will only be stored at an encrypted server at the University of Copenhagen.*<br><br>*I need an informed consent by you (which I will ask about in a second). For legal purposes, the data responsible person at University of Copenhagen will keep this*	Information

informed consent for five years. Therefore, the audio tapes will be stored at a secure and encrypted server at the University of Copenhagen. If you decide before that, that you do not want to be part of this interview, you can contact me and I will make sure, that your interview materials are deleted.<br><br>*I want to stress that this conversation is confidential, and that your name and any other information that could identify you will not be disclosed to anyone outside the working group of the project. Anonymous quotes from our conversation can be used in academic presentations, scholarly publications, papers for professionals or a wider public, or in connection with educational/teaching activities.*<br><br>*The research project has been approved by the Institutional Review Board of University of Copenhagen.*<br><br>----<br><br>Now, I would like ask about your consent to participate in the interview and to have it recorded [if no to any of the following, the interview will be terminated]<br>- Was the description of what you consent to clear to you?<br>[if no: read the description again]<br>- Do you agree to the interview being recorded and transcribed?<br>- Do you agree to the audio file of the recording being shared with the data responsible researcher at the University of Copenhagen?<br>- Do you agree to the anonymized transcripts of the interview being shared with Norwegian/Swedish researchers on this project?<br>- Do you agree to The University of Copenhagen being data responsible and keeping the audio files at a secure server for five years?<br>- Do you agree to the use of anonymized data from the transcription of the interview in this and future research projects and teaching?<br><br>Thank you.	Consent
**Part 1: Background and current (behaviour) work situation**	
Please begin by briefly describing how you started working professionally with dog behaviour and training [if not mentioned, prompt for professional background and current professional role].<br><br>For how long have you been seeing SRP cases?<br><br>How often do you currently see cases of dogs with SRP?	Informant background and current situation in terms of advising dog owners<br><br>Categorizing informants into one or more of vets/behaviourists/trainers<br><br>Informant's level of experience with SRPs and possible specialization with this problem
**Part 2: Defining SRP**	

What are the symptoms that owners notice and approach you with regarding SRP?<br><br>How would you define SRP? What are the typical symptoms?<br><br>Would you say that SRP is one thing or different things? For example, do you think SRP can have different motivations, e.g. anxiety, lack of learning, frustration, boredom?	Categorizing different types of SRP
**Part 3: Defining successful and not successful treatments**	
This project is about trying to identify factors that may differentiate successful and not successful courses of SRP treatment. So first, I would like to ask you how you would define a successful treatment of SRP. When would you say that you reached this?<br><br>And similarly, how would you define an unsuccessful treatment of SRP?	
**Part 4: Successful SRP treatments (what worked well)**	
Thinking back on your work with SRP dogs and their owners, please think of **one particular** case where the course of the treatment worked really well.<br><br>What in particular worked really well in this case?<br>[can be followed up with the following]<br>- how did the owner influence the course of treatment?<br>- how did the communication with the owner and owner compliance influence the course of treatment?<br>- how did the particular dog influence the course of treatment?<br>- how did the methods you used influence the course of treatment?<br><br>How has other courses of successful treatment been in relation to this case?<br>- what has been the same?<br>- what has been different?	Finding possible patterns in SRP treatments that work<br><br>Identifying levers<br><br>The aim is to get the informant to talk about both dog and owner and training method.
**Part 5: Not successful SRP treatments (what wasn't working)**	
Thinking back on your work with SRP dogs and their owners, please think of **one particular** case where the course of the treatment did not work as well.<br><br>What in particular was not working in this case?<br>[can be followed up with the following]<br>- how did the owner influence the course of treatment?<br>- how did the communication with the owner and owner compliance influence the course of treatment?<br>- how did the particular dog influence the course of treatment?	Finding possible patterns in SRP treatments that do not work.<br><br>Identifying challenges<br><br>The aim is to get the informant to talk about

- how did the methods you used influence the course of treatment?<br><br>If you have had other cases with less successful courses of treatment, how were they compared to this case?<br>- what has been the same?<br>- what has been different?	both dog and owner and training method.
**Part 6a: Current strategy/strategies for SRP treatment**	
Could you give me a brief overview of your typical treatment process for SRP? For example, how do you begin the treatment process, how do you typically proceed in the treatment process and at what point do you typically finish the contact with the owner?<br><br>Are there SRP cases where your approach is very different from this? If so, how and why?<br><br>What is your experience of using food in the training of SRP?<br><br>Why do you choose to use it/not use it?<br><br>If you choose to use food as part of the training, how do you advise the owner about it?<br><br>[depending on the answer to the first open questions, the following follow-up questions can be used]<br><br>How do you usually meet with the dog and owner?<br>[if not mentioned, prompt for online or physical meetings; at clinic/office or in owner's home]<br><br>How often and for how long do you usually consult with the dog and owner?<br>[if not mentioned, prompt for average frequency, duration and number of consultations and if this varies during the treatment period]<br><br>For the last couple of cases that you have worked with, please explain what you did in practice [if not mentioned, prompt for:]<br>- How was the training of teaching the dog to be left alone carried out?<br>- Were there specific things the owner had to do, when leaving the dog, and if so what were they?<br>- How often and for how long did you advise the owner to train?<br>- Were there other things that you advised the owner to train or do during the course of the treatment, and if so what were they?<br>- How did you advice the owner about alone time during the course of treatment?	Mapping out currently preferred treatment strategies
**Part 6b: Non-training treatment methods**	

Depending on who you ask, training is not always enough to treat SRP in dogs.<br><br>What do you think about medication for dogs with SRP?<br><br>What do you think about the importance of possible physical problems in dogs with SRP?	Mapping out attitudes towards non-training methods amongst different groups of professionals
**Part 7: Recurring dog characteristics and treatment adaptations**	
Now we will discuss your general experiences and observations regarding the background of dogs that come to you with SRP.<br><br>Are there any recurring characteristics or past life events that you frequently notice in dogs you treat for SRP? Referring, for example, to breed, age, genetics, socialization, early upbringing environment, other problematic behaviors, the amount of stimulation the dog receives, the owner, or anything else.<br><br>Does the dog's and/or the owner's background influence your treatment strategy? How?	
**Part 8: Owner management, expectations, compliance**	
Since the owner is such a big part of treating SRP in dogs, I would like to hear a little bit about your experience with owners.<br><br>What are the typical expectations of owners in terms of the treatment process and outcome?<br><br>In your opinion, how do you best help owners through a course of treatment?<br><br>In your experience, how do owners manage to live with the SRP problem?<br><br>And how do they manage the treatment protocol?	Expectations<br><br>Compliance and management<br><br>Owner experience
**Part 9: Information acquisition**	
Do you ever / or have you ever drawn on information about what sort of methods work in SRP treatment?<br>I am thinking of any kind of information, e.g. from books, online resources, scientific studies, evidence-based knowledge. Could you clarify how and when you use (or used) it?<br><br>Do you talk with other professionals, e.g. other SRP consultants, about what works? Are you involved in a professional network focusing on this?	What knowledge resources are used (if any)<br><br>Textual resources<br><br>Human networks

Quotes divided by themes

Theme 1: Co-occurring behavioural difficulties

Other fears

"Or a dog, [...], who has inner worries and is not feeling well." ID1SWE

"My experience is that it is very relevant for dogs with separation issues, that it's not just one thing but there are other comorbid diagnoses. Now, I don't make diagnoses according to my training, but there are comorbid problems that contribute." ID3SWE

"Those who come to me have dogs that panic and are scared and have several problems at the same time." ID3SWE

"[...] it is many who come to me who also have issues with car rides, and that's because these dogs worry a lot about what is going to happen." ID3SWE

"But also other stress-related behaviour, like increased resource guarding, and... Uh... starting to react during walks." ID3DK

"There are many different varieties. The ones I work most with are related to anxiety, but it can also be boredom and attention-seeking behaviour." ID1NO

"But if a dog has issues in several areas and that's not unusual really, I would say, then that affects the separation training too, and it has to do with all the impressions that need to be processed [by the dog] and then suddenly [the dog] has to be alone with all - everything it has experienced, in a way." ID4NO

"Then something that I find very interesting. It's when there's, like, a comorbidity between lots of other emotions that are out of balance, like a fear of sounds, and that's when I often see a lot that they lie and listen for that sound inside." ID1SWE

"Yes, I believe [...] that it can be a fear of sounds behind it that makes them more insecure or afraid of being left alone." ID2SWE

"Dogs that are afraid of sounds, I often think, can also have separation anxiety, and as for what depends on what is-, was it sound-sensitive and then got scared when it was alone or just found it difficult to be alone and then someone was drilling or fixing something?" ID2SWE

"So there are some of them...who also have other forms of anxiety. I mean, towards strangers or towards sounds. But definitely not all of them." ID2DK

" Whether it's pain, whether it's actual fears of sounds and such things. So all these things play a role." ID2NO

"A lack of understanding that if the dog has difficult periods, [...] right after New Year's Eve where it might be a bit more anxious. I mean, there's quite a lot of that." ID5NO

"I mean, it doesn't have to be separation anxiety to start with, it can be something external that makes the dog suddenly feel insecure about being home, like typically construction nearby, thunder...yeah." ID1DK

"Then it can be negative experiences." ID1SWE

"Or I have some cases when I've worked with dogs that really want to go out, but not to their owner, they just want to get out of the home. And sometimes it's because of negative experiences." ID1SWE

"And then there are some... who could be a little alone at home, where then something happens, and then they can't be alone at home anymore." ID2DK

"So I've had some, where it's the classic scenario, that they have been alone at home, where something unpleasant has happened, so to say" ID2DK

Early emerging response patterns

"[...] they have all received advice before. In most cases, it amounts to forcibly leaving the dog until it gives up. [...] Some never give up, about 20-25% never give up, and those are probably the ones who come to me because they don't give up no matter how long you leave them." ID3SWE

"sometimes you just get the feeling, when a dog comes in...it's just naturally nervous, right...It's the same when we get them for their first vaccination, and you can just feel, phew... 'and it comes from a good breeder too'. Yes, but uh... and it might also be that I have done a lot of work to socialize it, but it's just a sensitive nature, right. It's a feeling you can have. I can't say it more precisely." ID1DK

"There are some that have had it [an SRP] from the time they were puppies, who have never been able to be alone. Never have been able to be alone at home, where owners say that from Day 1 they have never been able to leave [the dog], even just to go to the toilet, and that they have never been able to leave, go out the door for even 10 seconds, so they have never felt that they could train anything at all related to [the dog] being alone at home." ID2DK

"So yes, then there are - some of them are those, where the owners have never been able to - where they have never been able to be alone. Where it has never been successful for the owners, right from the start." ID2DK

"Where - and I actually have quite a few of those. Those poor owners, they uh... bring the puppy home and do EVERYTHING 'by the book', right? They make sure there is peace around it. They...just go in and out of the door, or out to... the mailbox or something like that in that style and those puppies, they just go into a coma (note: become very passive), from the start. Yeah (...) And there I think - that - people are completely, that they have done something wrong. where you just think: "no, I don't believe that. I just think that you've been really unlucky." ID5DK

"and have gotten a puppy that can't. But, but it sounds like you've done everything right." ID5DK

Unmet needs

"I think that boredom in the form of, like, an overall under-stimulation, I often find it more a contributing factor rather than a cause in itself." ID1SWE

"As for stimulation that we touched on, that was it, specifically dogs that are very under-stimulated or wrongly stimulated, and that it's often a contributing factor. But the conditions for

being able to rest and take it easy don't exist because they want to play like a wound-up Duracell bunny when you close the door. That in itself, those experiences then become these negative experiences that contribute to the development of separation-related problems where that stimulation can be a... What's it called in Swedish?" ID1SWE "Contributing factor?" Interviewer "Yes, or ignite, that it kind of ignites the flame on the whole thing." ID1SWE

"That they are under-stimulated makes it difficult for them to relax [...]" ID2SWE

"Also- definitely, we can have frustration-related separation problems that come from the dog being under-stimulated and not panicking about being left. [...] But those who come to me generally do not have dogs that are under-stimulated and frustrated." ID3SWE

"[...] or that they are under-stimulated in general have a hard time relaxing because they, they have a lot of surplus energy or, or even find out that when they are alone, they can rampage freely and break into the trash cabinet or [laughs] whatever they want to do, or chew up the couch because they are simply bored and can do whatever they want in that situation." ID4SWE

"And then I have some [dogs] ... where it's not really separation anxiety, but where they are under-stimulated and so when they are alone, they find a lot of things to do because they have a hard time relaxing in that situation." ID4SWE

"Yes, so therefore I have the impression - well yes, so there are also those, where one has the impression, that - that it's part of it, that they can't relax. Where it seems like, they simply have a hard time settling down too, so it's not only - and this is my interpretation of it - that it's not only an anxiety about being alone, but also a, uh... lack of ability to settle down. I mean, they are frustrated over (...) they can't figure out how to settle down because they generally have a hard time settling down." ID2DK

"And then there are some, which I see, that it...that they are also used to, the owner being available, when they want - I mean, when they need it. When the purpose of the owner's presence is entertainment. So I also see it as some kind of frustration over, the owner disappearing. So it's not only, uh...yeah. And there you typically see a difference, when the owner is home, that these - some of the dogs, they can easily...they can easily lie by themselves and relax somewhere in another room from the owner. And there are some of the dogs, which seek the owner's attention, uh... all the time and have a very hard time...to...or at least show signs of significant unrest, if the owner ignores them, or if the owner doesn't give them the attention. They also show unrest, if the owner gives them attention, because then...yeah" ID2DK

"There are many different varieties. The ones I work most with are related to anxiety, but it can also be boredom and attention-seeking behaviour." ID1NO

"But it can also just be boredom." ID2NO

"It's really on the large and small scale, but I feel that many large dogs are those dogs that are- They don't get the attention and the, what shall we say? Activity level that they maybe need and want, and that they- That they maybe don't get to use their brains enough. So I think all those Border Collie mixes and all that. It's- They are so difficult." ID3NO

Theme 2: A body in flux

Early developmental shifts

"And then also, I think, I experience at least, that it can also be developmental periods. Partly when (note: transitioning from) puppy to young dog, [...]" ID2SWE

"But I see a connection between [SRP s] and, somehow, developmental periods. Often when the puppies - the young dogs - are in an easily-spooked period at 6-8 months, it can happen that suddenly they can't be left alone (even the young dogs). It’s when they are 18 months - there somewhere - [that] they usually develop problems." ID2SWE

"When it has started sexually maturating physically, it also becomes much more sensitive and sensitive to different things. That's where we see many dogs become... they are sold, euthanized, or..." ID4DK

"Oh... on age, I can actually see that it's often young dogs. So I have a bunch of puppies, and I have a bunch of dogs right around sexual maturity. [...] I definitely often find that I have these young dogs, that it's around sexual maturity. And some of them - sometimes the scenario is such – they could be alone at home for 4 hours and suddenly it can't." ID4DK

"Yes, but also around teenage. Because why are there some puppies that actually...seem like they're totally okay? There are also those, where I call them, uh... latent...like having latent home alone problems. I have those too. Where the puppies...cope with it, and then they reach sexual maturity, and then they can't. And then you've heard things like: 'Well...I've left a meat bone, or I've left something for it to eat, it only ate when I came home, right?' And then I think, yes okay so it hasn't been super comfortable with it, but it has still been able to accept it... and then something happens around sexual maturity, and then it just goes completely wrong. But I've also had puppies...where people say: 'well, it has been lying and sleeping soundly'. They have had video surveillance on. It has been lying and sleeping (...) And then it reaches sexual maturity, and then it just can't. So you think....Okay, what happened there? So why...why is it that some of them, when it's around sexual maturity, suddenly - I know that the brain is under reconstruction...but what's going on? I mean." ID5DK

"Yes, I also think around the age of one to two when they start to become adults and maybe start to develop a bit more of the normal adult behaviour and maybe become a bit clearer on requirements in terms of activities and such, and maybe then it starts to show a bit that owners have lost a bit of control over them. If they didn't get the training they needed. They start to become a bit big and strong. But that's in the one- One to two-year age." ID3NO

"But what I also see is the not understanding for example that a young dog is changing, so something that goes well one day might actually not go well the next day." ID5NO

Senior challenges

"When I look at the cause, it ranges from an older senior dog with declining senses or a medical condition." ID1SWE

"Absolutely, I think it does [physical problems play a role] and especially older dogs. Absolutely and especially the older ones, where there are several functions that have slowly begun to deteriorate." ID1SWE

"[...] and also in older dogs. Then, of course, since we know that it can be linked to cognitive dysfunction or dementia and similar conditions." ID5SWE

"Uh...older dogs." ID3DK "Yes, so you have a little bit older dogs that maybe start to have a bit of reduced vision and hearing and that type of thing. I think those things play a role." ID3NO

"And then again, I think when they start to get a bit stiff and sore and maybe begin to have reduced vision and such. 8 to 10, something like that. And then of course much older, and when you get to that there- I was about to say- The dementia phase right towards the end of life. Then we also tend to see that they become a bit more demanding." ID3NO

"That there is a senior dog can have a development. It can become more insecure because it feels changes in its body of various kinds." ID4NO

Health and behaviour

"Or a dog [...] who has pain issues or a medical condition and has an inner turmoil and is not feeling well." ID1SWE

"Dogs that have had a D-hip rating their whole life, that doesn't need to be the triggering factor. Unless they for some reason have developed a significant pain issue. But usually, I tend to see other stress behaviours as a result." ID1SWE

"At least I haven't found anything where they have looked into whether dogs with separation related problems often are in pain. [...]. So, the answer to that is, well, it's not the first thing I think about." ID2SWE

"Hugely important and absolutely underestimated (the importance of illness and pain) by everyone, including veterinarians I would say." ID3SWE

"They (physical health problems) do exist, though. I think they are less than, for example, with sound fear or aggression issues. [...] The ones that definitely make me wonder- I mean curious, are if there suddenly pops up a loneliness problem or leaving issue in the same environment where the dog has managed it for years before and I can't find a clear explanation. So. Absolutely." ID5SWE

"I attended a webinar, where one of their cases was a small dog, that...eventually turned out to have, I believe it was a slipped disc, and when they got the pain treated and under control, then it no longer had separation anxiety. I have no doubt that it's significant." ID1DK

"I feel that if it's related to pain, then it's... well, in that way, I don't think it's so much with those... separation anxieties. I believe it's more... those that...mmm... Those that react with a lack of trust towards people or other dogs, it's pain-rela - or not pain-related, but I find some kind of pain aspect in them." ID2DK

"Illness and pain, we just can't get away from it....It's just so essential." ID3DK

"And it ruins something in the training when you don't have the physical aspect with you." ID4DK

"But, but, but when those two things...that is the physical and the mental part, if they are in balance, then you have no problems. We're not just talking about being home alone, we're talking about - you have no problems in life. Nothing you can't solve." ID4DK

"Both chemical imbalances meaning there are other things in the picture. Whether it's pain, whether it's actual fears of sounds and such things. So all these things play a role." ID2NO

"I believe skin and stomach and joints and back and all that can play a role. Also, ehm, yes. Other medical problems." ID3NO

"I feel that a good number of these dogs get better just with treatment of joint issues." ID3NO

"If something is pain-related or, as I mentioned earlier, something like itching and allergies or something going on with the intestines or stomach, clearly, it affects the dog's response very much." ID4NO

"A lack of understanding that if the dog has difficult periods, for example, illness, [...] I mean, there's quite a lot of that" ID5NO

"The significance of (physical problems) is huge. It's complete- And there we have the chicken and the egg situation." ID5NO

"[...] but also that I with female dogs that are in heat can have, much more that they don't want to be alone. Depending on where in the heat they are and male dogs can howl and so because there are female dogs in heat nearby, so that it might not have anything to do with separation anxiety in that way." ID2SWE

Theme 3: Dog training gone wrong

Not trained

"that it absolutely is not used to this, has never been trained" ID1SWE

"And mainly this... it's huge, but it might not be about the dog, this that there hasn't been any training at all. Just expecting it to work" ID1SWE

"[...] and also that they are not trained in it." ID2SWE

"But it's also very common that they just haven't been trained to be alone" ID4SWE

"A common factor, or a quite common factor, is that they haven't even trained [the dog] to be alone when they seek help but have just realised [the need]. And especially during the pandemic, it was very common that they never needed to leave the dog and then suddenly the dog is 2 years old, and they realise that they might need to start training [the dog] to be alone and then they haven't laid any groundwork for it." ID4SWE

"It also happens, it's a bit related, but even worse than the first, as I said, there are also those who, who have tried to leave, the first example I'm thinking mostly about those who haven't left at all and not trained it, then we have those who have left and just thought it would work because now the dog is adult and then it should be able to be alone. And then they realize after a while: It doesn't work. The dog becomes more stressed or starts to destroy things or bark or so. So lacking, lacking training basically, not having trained the dog is the most common issue." ID4SWE

"Then I also think that we have seen an increase since the pandemic and that is really interesting because it really tells us all that it actually is a very big part about learning and socialization." ID5SWE

"[...] and ... how skilled they have been at teaching the dog to be - that is to rest by itself and be independent, right? As a young, as a young dog, right? How much has it, has one socialized it [...]" ID1DK

"It could be that the owner has been sick...and has been home for a longer time" ID3DK

"I mean, I think after corona, there have been these 20-25% more dogs. Uh...and there are many after corona has ended, who really didn't...catch up and [...]who really say, 'but I haven't, I really don't have time for the dog, now we're giving it back or to a shelter, right. [...] They say: 'Now we are home. Well then we can have a dog'. But they haven't trained being alone at home, right. They have trained 'we are home together all' - 'and now I - and now it's...now corona is over...now it's...now we're going to work, and then we haven't done...oops.' And then they say: 'then we can't keep the dog, because the neighbors they complain about'...down to the rescue center with them." ID4DK

"Everything from that I see very often dogs that have been with the owner a lot, like typical corona dogs. And I obviously see that if they- they do what they are used to, so if they are used to always being together, then it's clear that it will be a big shock to be separated" ID2NO

"If you think about it in terms of the pandemic, maybe (note: the dog) was very much together with the owner and being carried around and is on them all the time and such, that then gets the same type of problems." ID3NO

"There are some dogs that are rehomed, for example from a breeder, and then they have lived together with several dogs in that home. And you often get conveyed that: 'Sure the dog can be alone', but actually it has never been alone before. It has always been with let's say 4 other dogs in that home with the breeder.[...] It goes back to whether the dog has learned it. It might not have learned to be completely alone." ID4NO

"[...] we also get a lot of this, right, the companion dogs, who are totally unprepared to be alone [...]" ID5NO

"I see a lot of that, and then there's also something about the owners' perception of reality. It's quite obvious where there's a lack of learning. They just shove the dog in and are completely surprised that it can't be alone." ID5NO

"Generally speaking, it means that some of these, what should I say? Companion dogs that have very much- They have spent the first months of their life, then they had sat on the lap and been cuddled by the owner. And then suddenly they are supposed to be home alone. That- They are- Dogs that haven't learned during the first 2 months with the owner that they should- can be alone for short periods are overrepresented. One starts too late to address it [...]" ID5NO

Negative experiences during training

"that the training has progressed too quickly and practiced with entirely the wrong feeling during training" ID1SWE

"They've moved too fast." ID2SWE

"Owners often feel very guilty. And it's tough for them, of course, and they have all received advice before. Which in most cases is about forcibly leaving the dog until it gives up." ID3SWE

"And then, in addition, it comes down to whether the owner has left the dog early on. Like this: 'Oh, it's sleeping. Now I'll go.' And then the dog has had a bad experience [...]" ID2NO

"I would also say that one characteristic is extremely unrealistic [owner] perceptions, and that they get them from Dr. Google- Many get [ideas] like this: leave the room when the dog sleeps. Can you imagine anything more cruel than you go to sleep, and then everyone's gone when you wake up?" ID5NO

"The owner has taken time off (note: work) - three days, right? To train home alone, right? [laughs] Classic" ID3DK

Theme 4: Breed type

Breed and genetics are important

"Then I see certain typical, like the one I have myself, working dogs, that are very handler-bound, so to speak." ID1SWE

"But compared to the population that I typically see on the street, I think I see more of the small curly dogs. Like poodles and poodle mixes, especially the smaller varieties like Maltipoos. And I also see Miniature Pinschers quite a bit. Compared to how few Vizslas I see, I think I see a fair number of Vizslas. I should remember, there's probably something more that I'm not thinking of right now. Yes, Springer Spaniels, the Welsh ones. So generally, maybe. I think I see it more in the smaller dogs than in the larger ones, which makes it very hard to say if it's actually a genetics question or if it's how we..." ID5SWE "Know what it depends on?" Interviewer "handle them, exactly, it's very very hard." ID5SWE

"So it's very often small dogs. It's very often the small companion dogs. But it's super hard, because there are also really many of them right now, so it's...well, it's difficult to say anything about." ID2DK

"Companion dogs are bred to be together. They take it [separation] much harder. Interestingly, we have some breeds that aren't necessarily bred to be companion dogs but still have it [an SRP]. For example, we know the Dachshund can be very prone to SRPs. So yes, breed definitely plays a role." ID2NO

"And then there are the companion dogs. Those that are bred to be around our feet. It's typically harder for them to learn to be alone," ID4NO

"Then we have breeds that are more attached than others." ID2NO

"There is presumably some genetic factor also, that matters here in terms of anxiety at least" ID1DK

"I have this idea that there must be some genetics too - because I - some of these puppies, where you think, there's nothing on the breeder...when you talk about, what kind of litter is it, what kind of breeding? Then there's nothing there. There are no, uh... red flags that go up, at all. Where you just think: 'why can't that dog be alone at home?' And it's those, where it - where they almost can't do it from the start, or what?" ID2NO

"I believe breed absolutely plays a role," ID3NO

"Very much the social ones, those bred for companionship, closeness, and then hyper herding dogs [...]" ID5NO

"But specific breeds of small dogs I think becomes a bit more difficult." ID3NO

There are no breed related patterns

"As I've said about breed, I don't feel like I've directly seen that." ID2SWE

"I can say that the breed I've worked most with when it comes to loneliness issues is the poodle, but that doesn't really say anything. One could say poodle owners might be more responsible and seek help more? We know that people don't seek help for this as much as they should. So that... No, I don't see any patterns in this actually." ID3SWE

"Then I think it's quite spread across breeds. I'm not someone who feels that these always come with separation problems." ID4SWE

"What I can see, is that I can't see that there's any breed-specificity - I can't see that I have specific breeds." ID5DK

It’s what you do with the breed that matters

"Then I think that certain dogs of the breeds like Welsh Terriers, or a Lab or a Beagle that are a bit more inclined to enjoy being in large packs or the polar dogs. There, separation can be difficult, especially for the polar breeds, but it's greatly reduced just by having more dogs. But it's not so much about the human as it is just wrong that there's only one dog or somehow that's where the shoe pinches." ID1SWE

"And the last is probably Huskies that haven't been in sled training, who came as puppies to their owner. Not getting the stimulation they need. And then are left alone. And simply out of boredom wreck the house. Not necessarily separation problems. It's a very social breed so it might be some separation from being alone. So it's not necessarily separation from people, but just being alone is terribly boring. As soon as they get another dog, it's better." ID2NO

"It's really on the large and small scale, but I feel that many large dogs are the dogs that are- They don't get the attention and the, what shall we say? Activity level that they might need and want, and that they- That they maybe don't get to use their heads enough. So I think all those Border Collie mixes and all that. It's- They are so difficult. Lots of retrievers." ID3NO

"And it's probably also because we have a very wide range of breeds, so it's not just these working dog breeds, but we also get a lot of this, right, the companion dogs, who are totally unprepared to be alone and some of these hyper working dogs that when they first go berserk when they're alone, they really go berserk." ID5NO

"And here is especially, I think, these hyper working dogs, the herding dogs. They are absolutely in a class of their own. I mean, if you come with a 6-month-old Border Collie that you haven't taught to be home alone. Then you have a long road ahead" ID5NO

"But also dogs that want to go out to hunt, so those with a hunting drive. [...] when they finally get out, they head to the woods rather than start looking for their owners." ID1SWE

"I think genetics absolutely play a role, but I think there are very many environmental - type things that are at least as important, if not more." ID3NO

Theme 5: Changes to routine and environment

Changed routines

“Yes, I think it’s that suddenly things have changed, like routines. That they [the dogs] have moved, even if it’s with the same family that they have moved. That they [the owners] have divorced so that the dog just lives in one place with half the family, so to speak. And changed work hours [...] can affect it [the SRP]. A change in the family if they have had children – such things can also affect the dog.” ID2SWE

"Then there are some, where it happens, where the owner moves," ID2DK

"It could be, for example, uh... that people are about to have children or have had a child, uh... and... the mother has been staying at home. The child has, uh...it has been awake several times at night. So this disturbance in sleep rhythm...and not being allowed to sleep, because that's often where they get a lot of rest. So when there are... small children in the home. Uh...small children, who require a lot of attention, and uh...who disturb the nighttime sleep and the general peace, uh, in general [laughs]. Uh... there, I experience, uh... well, stress behaviour." ID3DK

"Uh...and changes in daily routines in general" ID3DK

"I also have older dogs (note: not puppies), where it's related to moving, for example, or something like that. Uh, they've moved from the countryside to the city..." ID5DK

"[...] the dog has been home alone, functioned fine, and then there have been major changes in the family. There have been divorces, house changes, suddenly there's been renovation in the neighboring house. So other external reasons that suddenly have created major changes for the dog which then has triggered this." ID2NO

"That a death of a dog or a human can indeed affect. And illness in a family over time. Serious illness that means the dog has received little training. It becomes a sort of human factor. Absolutely, of course, very understandable then. That there's a change." ID4NO

"Dogs that lose their...friend, another dog in the home maybe." ID3DK

Rehoming

"Then I see it a lot with rescue dogs and or rehomed dogs in general." ID1SWE

"I see connections with these rescue dogs or rehomed dogs, as I mentioned, that bond very closely with their owners and find it difficult to be away from them." ID1SWE

"No, but as I said before, with rehomed dogs I experience it and those who have either changed homes within Sweden or that are former street dogs or rescue dogs often need help, or time to settle." ID2SWE

"I have a disproportionately large number of rescues, in general, that come to me, but that’s where we also see- I experience that I see more separation-related problematic behaviours." ID5SWE

"so there are some, where you don't really know if it has been there before, but where it is at least very clearly seen, when the dog has been rehomed." ID2DK

"That thing with, that they have been...relocated. That is, another home." ID2DK

"And of course, uh...street dogs...relocation dogs...yes, that's like, uh...can one say, must one say, that there... one must take into account, that it, uh...comes, usually." ID3DK

"I have also had owners who have received rehomed dogs for example. Who have found them to be a bit more challenging than what they perhaps expected." ID3NO

Leaving the breeder

"Early separation from the mother dog, or if a puppy became sick and needed to be removed or the mother dog got sick and was removed. Or the last puppy left in the litter who has been with the mother dog a lot, there I can see a certain tendency." ID1SWE

"Well, uh...separation from mother and siblings." ID3DK

"Well, there's something about the time when they come to a new home from 8 weeks of age, that very much is conducive for learning then to be alone. So can- I see with some dogs that when they have lived a long time with the breeder, for example until they are 4-5 months, that it then is more troublesome for them to learn to be alone." ID4NO

Theme 6: Psychological interplay between dogs and their owners

Owners are stressed

***Editorial note**: Within this subtheme, participants articulated a professional understanding of separation-related problems that extended beyond the dog to encompass owners' emotional wellbeing and life circumstances. Accounts frequently positioned owner stress, anxiety, or relational strain as relevant to the dog's difficulties, reflecting how practitioners may interpret canine behaviour through the lens of human psychosocial stressors. We note, however, that some formulations (e.g. ID1DK) may be interpreted as gendered, as they explicitly attribute anxiety-related concerns to female owners rather than to owners more generally. Such statements are included to document the range of professional perspectives expressed in the dataset, while the gendered framing was not part of the coding rationale nor the analytic claim.*

"Young girls with diagnoses, anxiety diagnoses. We have had a couple of cases where we discussed whether it's the dog that has separation anxiety, or if it's actually the female owner." ID1DK

"Then there are really many owners who say that they themselves have anxiety. But I don't know if that's just because there are really many people in today's Denmark who have [laughs] anxiety. I dare not say, but...it's not unusual at least." ID2DK

"I also experience... when I start talking about stress in the dog, then they say 'Oh no, I know what you're talking about because I've been off work sick with stress.' So, I often experience that when we start to get into it [the dog's stress], then the owner has also often been... exposed to stress, and has maybe had a breakdown due to stress." ID3DK

"I have a little gut feeling theory that if you leave the dog while you're very anxious yourself, then the dog senses this." ID2NO

"it is, uh...many of them, I've had, where... that there has been high separation anxiety (...) I've found out along the way in the process, that it's actually a couple that is on the verge of breaking up" ID3DK

Owners lack competence

***Editorial note**: This subtheme gathers excerpts illustrating how participants suggested that perceived deficits in owner competence regarding dog care may contribute to SRPs. One excerpt (ID4DK) contains an explicitly gendered and stereotyping characterization. We retain the excerpt verbatim for transparency, quoting it directly from the interview transcript; however, it is not foregrounded in the main findings. During analysis, the first author coded this excerpt within the subtheme for its account of perceived owner competence as a contributor to SRPs. The gendered characterization was not part of the coding rationale and is not part of the analytic claim. We flag this explicitly to contextualize the excerpt and mitigate the risk of gendered discreditation.*

"how well-functioning the owners are. Or what can we say...their competence? Yes, their owner competence, yes, we can call it that." ID1DK

"And then there are the women. The women have enormous gaps (of knowledge?)... and are not continuous, and they eh... they come in, and kind of eh... they don't see that it's a dog. But they think a bit like it's a baby eh..." ID4DK

"But that they don't expect to have to spend a lot of time on them [dogs], I think that's a recurring theme. That people had a bit of a wrong understanding of what they were getting into when they got a dog." ID3NO

"Yes, and right, unrealistic owners." ID5NO

Dog-owner interaction patterns

"Yes, it's an owner factor. Yes, the owner factor plays a significant role. I think they do. Partly how strongly they attach to the dog [...]" ID1DK

"So it also has to do with the person's age, because if you are in your thirties, and things just have to work, so uh... then you get involved in things and want it to work. But if you're young, and you haven't had a child yet, then you think you've gotten a baby, and then that's where it goes wrong. And the older ones [...talking about themselves] you're retired, or the kids have moved out. And then it's those who fare the best, it's those here [signals middle], because they need so much structure in their lives already." ID4DK

"I absolutely believe personality can affect the dog, how bonded it is to its handler." ID2SWE

""[...] there are some [cases] where [...] the dog is obviously very attached to one owner, where there's a big difference depending on whether it's one owner leaving [...] or the other owner, and if one owner disappears from the home, then the dog also howls." ID2DK

"There are some that are just happy, as long as there is another person, and then there are those who...uh...where - if the dog needs to be looked after (note: by someone else), it also howls for 5 hours and wanders around...so it just shows - that it needs to be the owner who provides that security, and another person isn't enough. And then there are those for whom it's perfectly fine if there's another dog." ID2DK

"The dog has been sick, yes. The dog has been sick, uh...and received a lot of medication. And, uh...cuddled a lot. It's not because the dog shouldn't cuddle, but this...hyper-attachment, it has

become, is like - a dependency, uh, I think more, it's like dependency - because...we know that there are attachments, and we know that we form attachments with our animals." ID3DK

"And I don't know if this answers the question, but I also believe this mismatch between owner and dog and the time they have spent on training and how dedicated they are to the dog, I think plays a very big role." ID3NO

Additional note: no recurring traits

“No. We think, well, it can be anything.” (ID1NO)
